# The treatment experiences of women with perinatal OCD on Mother and Baby Units: qualitative investigation of the perspectives of women and professionals

**DOI:** 10.1192/bjo.2026.10976

**Published:** 2026-02-10

**Authors:** Ella Davenport, Vanessa Lawrence, Fiona L. Challacombe

**Affiliations:** Institute of Psychiatry, Psychology and Neuroscience, King’s College London, UK; South London and Maudsley NHS Foundation Trust, London, UK; Department of Health Service and Population Research, Institute of Psychiatry, Psychology & Neuroscience, King’s College London, UK; Department of Experimental Psychology, University of Oxford, UK

**Keywords:** Obsessive–compulsive disorder, perinatal, mental health, Mother and Baby Unit, qualitative research

## Abstract

**Background:**

Perinatal obsessive–compulsive disorder (pOCD) is a common mental health difficulty. For some women with pOCD, a psychiatric in-patient admission is deemed necessary. In the UK, Mother and Baby Units (MBUs) are currently best practice for in-patient admission in the perinatal period. Wider OCD literature and pOCD case studies suggest the MBU environment may pose challenges to the treatment of pOCD.

**Aims:**

To date, there has been no research exploring pOCD on MBUs, therefore, this study aimed to qualitatively explore women and professionals’ experiences of pOCD on MBUs.

**Method:**

Semi-structured interviews were conducted with eight women who self-identified as having experienced pOCD and an admission to an MBU, and ten professionals who had experience working with women with pOCD on MBUs. Interviews took place virtually and were recorded and transcribed. Reflexive thematic analysis was used to analyse the data.

**Results:**

Six themes were identified. (a) ‘MBU a last resort for OCD’, (b) ‘Developing a shared understanding of OCD’, (c) ‘A whole team approach to treatment’, (d) ‘Choice and control over exposure’, (e) ‘Ward as a safety net’ and (f) ‘Transitioning back to real life’.

**Conclusions:**

The research highlighted a number of challenges in providing treatment for pOCD in this environment and suggestions are made for the development of clinical guidelines for supporting women with pOCD and designing specific training for MBU professionals.

Although not a formal diagnostic specifier, obsessive–compulsive disorder (OCD) that is triggered or exacerbated during pregnancy or after childbirth is commonly referred to as perinatal OCD. Perinatal OCD (pOCD) is a common mental health difficulty, with an estimated point prevalence of 2.9% during pregnancy and 7.0% postpartum,^1^ both of which are higher than the population prevalence of OCD which is estimated to be 1.2%.^2^ Estimated rates for newly onset OCD in the perinatal period range from 2 to 4%, with exacerbation in 8–70% of women with pre-existing OCD.^3^ Women^a^ experience obsessions and accompanying rituals/compulsions typically relating to their baby and caregiving tasks. This can present in different ways; for some it may include thoughts of accidental harm coming to their baby through contamination (contamination OCD) or negligence (checking OCD), whereas for others this may include thoughts of deliberately harming their baby (rumination OCD). This can be extremely distressing for women,^4,5^ and their support network, who may try to accommodate OCD through partaking in rituals, adapting routines and facilitating the avoidance of triggers.^6,7^ The severity of pOCD can vary, and for some women, compulsions intended to manage their distressing thoughts can raise risk issues, including child safeguarding concerns. For example, a woman with worries about contamination may not allow her baby to have contact with others, including healthcare professionals.

Guidelines in the UK recommend all people with OCD are initially offered cognitive behavioural therapy (CBT) with exposure and response prevention (ERP),^8^ which has an effect size of approximately 1.3 compared with wait-list controls.^9^ Medication is also a recommended first-line treatment for OCD, and for individuals where OCD is causing severe functional impairment, CBT should be combined with medication.^8^ ERP can be extremely challenging for individuals, as a result of the high levels of fear and distress associated with exposure and this can lead to CBT refusal and drop-out.^10^ Additionally, healthcare professionals can be apprehensive about ERP, and worried individuals will not cope with the emotional distress of exposure even outside the perinatal context.^11^ Engagement in ERP can be supported by the therapeutic alliance, and a clear agreement and understanding of therapy tasks.^12^ Research suggests CBT is an effective community treatment for women with pOCD, particularly when delivered intensively, in shorter time frames.^13,14^

In-patient admission is necessary for some individuals with OCD, owing to the severe levels of functional impairment that can be involved, risks associated with suicidal ideation, co-morbid mental health difficulties and self-neglect. Currently, there is no national data available on the number of individuals requiring in-patient admission for OCD; however, international data suggest low admission rates.^15^ Within the UK, there are very few specialist in-patient or residential settings specifically for individuals with OCD.^16^

For women in the perinatal period, admission may also be necessary because of the risk to their baby, usually owing to the impact of OCD. Treating OCD on general psychiatric in-patient wards can be challenging, because of rotating staff teams with varied awareness of OCD.^16^ The ward environment, with reduced responsibility and potential reduction in triggers, can also result in temporary improvement of OCD symptoms, with symptoms returning after discharge.^16^ Specialised OCD treatment environments (e.g. residential settings) can be effective for severe or treatment refractory OCD; however, these environments do not currently allow for co-admission of women and babies.^17^

Mother and Baby Units (MBUs) are currently best practice for in-patient admission in the perinatal period.^18^ Women with a range of severe and complex mental health conditions are admitted alongside their babies to support maternal mental health and the mother-baby relationship. Women can be admitted during pregnancy or postpartum and stays are typically weeks or months. In the past decade, MBU service provision has expanded across the UK. There are currently 22 MBUs in the UK, with varying capacity (range of 4–12 beds).^19^ However, there remains a paucity of beds in some regions of the UK,^20^ therefore some women requiring in-patient admission in the perinatal period access this on general acute psychiatric wards, where they are not admitted with their babies. MBUs have been established in several countries across the globe (France, Germany, Australia, India, Sri Lanka and the USA). There are international differences in the structure, possibly owing to differing healthcare models (e.g. privatisation versus national health service, national financial resources and investment in mental health services.^21^ Despite recent advances in MBU development, global provision remains sparse, therefore many women requiring in-patient psychiatric admission in the perinatal period may continue to receive care on general acute psychiatric wards. Research has found that professionals on the MBU play a large role in women’s experience of admission to the MBU, and women value the perinatal expertise of staff and the kind, compassionate stance professionals take.^22^

At present, there is no national data on presenting problems but women with pOCD are likely to constitute a low percentage of overall admissions to UK MBUs.^23,24^ Because of the range of mental health difficulties and risk presentations, MBUs must have procedures in place to manage risk.^25^ This is potentially challenging in the context of OCD presentations, as women with pOCD are likely to need a different approach to risk management. Challacombe and Wroe^26^ describe the cases of two women admitted to MBUs after experiencing intrusive thoughts of deliberately harming their babies. Prior to admission, pOCD was not recognised and the women were viewed as ‘at high risk of harming their children’. On the MBUs, mother-baby contact was reduced, and child protection proceedings initiated. The cognitive model of OCD suggests there is no risk of women acting on unwanted intrusive thoughts in this context,^27^ and research has found no association between intrusive thoughts and increased risk of infant harm.^28^ Therefore, limiting mother-baby contact and raising safeguarding concerns was unnecessary, and reinforced women’s beliefs that the thoughts mean they will harm their baby, thus maintaining OCD and exacerbating distress.

While case literature suggests there may be challenges associated with treating pOCD in the MBU environment, to date, there has been no research specifically exploring women’s experience of admission and treatment, nor healthcare professionals’ (HCPs) experience of supporting women with pOCD on MBUs. The current study aims to address this gap in the literature.

## Method

### Ethical approval

Ethical approval was granted by Yorkshire and The Humber – Leeds West Research Ethics Committee (ref: 23/YH/0118) and the National Health Service (NHS) Health Research Authority. The authors assert that all procedures contributing to this work comply with the ethical standards of the relevant national and institutional committees on human experimentation and with the Helsinki Declaration of 1975, as revised in 2013.

### Study methodology and setting

This study is a qualitative interview study using reflexive thematic analysis. The research was conducted in the UK, within which there is the NHS). The NHS provides government funded healthcare services to UK residents, which includes funding in-patient admissions to MBUs.

### Theoretical underpinnings

The study was grounded within a critical realist ontological position and the epistemological stance taken was contextualism. In this position, OCD is conceptualised as a definable mental health condition; however, individuals’ experiences are influenced by contextual factors.^29^

### Participants

#### Service users

Inclusion criteria specified that service users should have experienced pOCD and admission to a UK MBU within the last 3 years. Service users self-identified as having pOCD. There were no inclusion criteria relating to age or gender. Service users currently on MBUs were not eligible.

#### Healthcare professionals

Inclusion criteria specified that HCPs should have experience working with service users with pOCD on a UK MBU within the last 3 years.

### Recruitment

#### Service users

Service users were recruited through social media and lived experience led OCD networks. Information about the study was also shared with service users who met the inclusion criteria via clinicians working on an MBU in the Southeast of England. Effort was made to recruit service users with experience of a range of MBUs, and with a variety of OCD presentations (e.g. contamination, rumination or checking OCD).

#### Healthcare professionals

HCPs were recruited through advertising on social media. The opportunity was also shared with clinicians working on an MBU in the Southeast of England. HCPs from a range of different professional groups with differing amounts of clinical experience on MBUs were recruited, and effort was made to recruit HCPs from a range of MBUs.

#### All participants

Participants expressed interest via email. They were provided with an information sheet and telephone call to explain the nature of the study, answer any questions and determine eligibility. Written informed consent was then taken. Nine service users and 12 HCPs contacted the research team, and of these, 8 service users and 10 HCPs took part in the research. Reasons for not participating included no response to email (*n* = 2) and difficulty determining eligibility for one professional. Recruitment continued until information power was reached^30^ and the data was judged to have sufficient detail, meaning and nuance. Preliminary analysis was conducted alongside data collection.

Following interviews, all participants were also asked whether they knew others who may be interested in taking part.

### Procedure

#### Patient and public involvement

The study design and procedure were presented to a group of perinatal individuals with lived experience of perinatal mental health difficulties, some of whom had also experienced MBU admission. The group highlighted how challenging it may be for perinatal individuals to share distressing experiences, therefore an optional follow-up call was added to the procedure. Topic guides and interview procedures were developed collaboratively with a woman with lived experience of pOCD and MBU admission. She was paid £20 per hour for her involvement.

#### Consent and demographic questionnaire

All participants completed an online consent form on the day of the interview. Participants then completed a brief demographic questionnaire regarding their age, ethnicity and gender.

#### Interviews

Interviews were structured around topic guides, with separate guides for service users and professionals (see Supplementary material for topic guides, available at https://doi.org/10.1192/bjo.2026.10976). The topic guides included questions about the experience of admission and discharge from the unit, staff understanding of OCD, specific treatment accessed/offered on MBUs and general suggestions for improving MBUs for service users with pOCD. The topic guides were used flexibly and reviewed throughout to consider revisions to the wording and structure. Following the interview, the interviewer went through the debrief sheet. At the end of the interview, participants were offered the option of arranging a follow-up call for the next day. Participants received a £20 gift voucher for their participation.

#### Analysis

Interviews were recorded, transcribed verbatim and anonymised. Data were analysed using reflexive thematic analysis,^31^ with NVivo software, version 14 for Windows (Lumivero, Denver, Colorado, USA; https://lumivero.com/products/nvivo/). Data from HCPs and service users were analysed together in order to explore different perspectives on the same themes.

Braun and Clarke’s^32^ six phases guided the analysis, as detailed in Table 1. Data from professionals and women were analysed together to explore different perspectives on the same themes. Transcripts were thoroughly read and checked against the interview recordings. Initial ideas and areas of interest were noted in the transcript margins. Each transcript was then re-read and coded by E.D., using semantic and latent codes. Transcripts were read by the research team, and to further support reflexivity and identify other possible interpretations, 4 of the 18 transcripts were also coded by F.L.C. and V.L. Different combinations of codes and sub-themes evolved through discussion between the research team. These were refined and drawn out into named themes. Though the interviews were informed by pre-determined topic guides and the researcher’s own positioning, analysis aimed to be inductive.

**Table 1:** Characteristics of women (*n* = 8)

Participant characteristics	Category	Frequency (*n*, %)
Age	18–24	1, 12.5
	25–29	1, 12.5
	30–34	2, 25
	35–39	2, 25
	40–44	2, 25
Ethnicity	White – English, Welsh, Scottish or Northern Irish	6, 75
	White – Other	1, 12.5
	Mixed or Multiple Ethnic Groups – Other	1, 12.5
Gender	Woman	8, 100
Location of Mother and Baby Unit	England	7, 87.5
	Scotland	1, 12.5
Length of stay	<1 month	1, 12.5
	1–2 months	3, 37.5
	3–4 months	4, 50
Primary OCD presentation	Contamination OCD	4, 50
	Rumination OCD	3, 37.5
	Checking OCD	1, 12.5

OCD, obsessive–compulsive disorder.

#### Reflexivity statement

Reflexive thematic analysis involves researchers reflecting on their positioning and how their background may have influenced the research.^31^ E.D. kept a journal to record thought progression and decision-making and reflected with the research team throughout the process. E.D. is a White British, female, trainee clinical psychologist with experience working with adults with OCD and on an MBU, all of which may have influenced her approach to interviews and analysis. Throughout the analysis process, E.D. reflected on how her grounding in CBT may influence the interpretation of the data. For example, using the term ‘safety behaviour’ within codes when this was not used by all participants and initially viewing reassurance from staff as incongruent with the CBT model. Reflecting with the research team supported E.D. to remain close to the data and consider different interpretations, including considering the unique environment of an in-patient ward and the severity of mental health difficulties service users are experiencing on the MBU.

While working on an MBU, E.D. met one of the service users prior to her taking part in the research. She was not directly involved in this participant’s care. E.D. had also worked with two of the professionals. She reflected with the research team about how these prior relationships may have influenced participants’ responses and her interviewing style.

F.L.C. is a clinical psychologist with experience working with perinatal individuals with pOCD and researching pOCD. V.L. is an experienced qualitative researcher, with limited knowledge of pOCD prior to the current study.

## Results

### Participant characteristics

Eight service users, all identifying as women, with experience of pOCD and admission to an MBU were recruited. They accessed six different MBUs. Seven women were admitted to the MBU with their baby postnatally, one woman was admitted in her final trimester. A summary of their characteristics is in Table 1.

Professional characteristics are shown in Table 2. HCPs were from a range of different clinical backgrounds. All had experience working on MBUs in England. Four participants reported receiving specific OCD training. Three were clinical psychologists, who received this as part of their professional training, and the fourth an occupational therapist who attended internal training at work.

**Table 2:** Characteristics of healthcare professionals (*n* = 10)

Participant characteristics	Category	Frequency (*n*, %)
Age	18–24	1, 10
	25–29	1, 10
	30–34	4, 40
	35–39	2, 20
	45+	2, 20
Ethnicity	White – English, Welsh, Scottish or Northern Irish	7, 70
	White – Other	1, 10
	Black or Black British – Caribbean	1, 10
	Asian or Asian British – Other	1, 10
Gender	Woman	10, 100
Professional title	Clinical Psychologist	3, 30
	Occupational Therapist	2, 20
	Mental Health Nurse	2, 20
	Social Worker	1, 10
	Psychiatrist	1, 10
	Nursery Nurse	1, 10
Experience working on Mother and Baby Units	<6 months	2, 20
	Between 6 months to 1 year	1, 10
	Between 1 and 3 years	3, 30
	More than 3 years	4, 40

### Findings

Six themes were developed from the data (see Table 3). Quotes are labelled with participants’ unique identifiers, which includes their participant group and reference number.

**Table 3:** Overview of themes and sub-themes

Theme	Sub-theme
1. Mother and Baby Unit (MBU) a last resort for obsessive–compulsive disorder (OCD)	Calls for earlier help in the community
	OCD reaching a crisis point
	Autonomy over the decision to be admitted
2. Developing a shared understanding of OCD	Sharing knowledge of OCD across the staff team
	Piecing together an individualised understanding of OCD
	Collaborative, transparent treatment plans
3. A whole team approach to treatment	Difficulty maintaining a consistent approach
	Taking responsibility for OCD treatment
4. Choice and control over exposure	Gradual, planned exposure
	Unexpected, ‘non-therapeutic’ exposure
	Meeting women halfway to manage distress on the ward
5. Ward as a safety net	Pushing with exposure experiments
	Reassurance of observations
6. Transitioning back to ‘real life’	Translating learning from MBU to home
	Plan for ongoing support
	Role of family in returning home

### Theme 1: MBU a last resort for OCD

#### Calls for earlier help in the community

Women expressed a preference for community support rather than in-patient admission; however, some women experienced difficulties in accessing this. Women wished they could have accessed support sooner and believed earlier intervention may have prevented their OCD symptoms from escalating, reducing the need for admission. Some women described an inadequate support offer from local community teams.

‘I think I needed way back to have people come and help me at home, that’s what I really needed and that’s what I asked for. I needed support and my family needed support, and that was not available - genuinely not available […] which is really why they put me in hospital.’ (W7)

Professionals echoed the importance of community intervention for pOCD, describing how ‘OCD is managed quite well in community teams’ (P10 – Social Worker). This contrasts the challenges women described in accessing effective community support. Professionals’ views may reflect a perceived rarity of OCD on MBUs, with professionals describing OCD as ‘not as common as the other disorders’ (P2 – Occupational Therapist).

#### OCD reaching a crisis point

There was a sense from women and professionals that OCD has to be severe for MBU admission, ‘to a point where they’re not able to function in their daily life’ (P10 – Social Worker). Women perceived their OCD to have reached a crisis point and were also experiencing co-morbid mental health difficulties.

Worsening OCD symptoms had a knock-on impact on women’s overall mental health. Women described experiencing depressive symptoms, ‘having a lot of suicidal thoughts’ (W5) and feeling as though intrusive thoughts were tipping into psychotic hallucinations.

In contrast, one woman described how she had not experienced suicidal thoughts, and as a result, professionals did not think she was in a crisis. She described feeling as though her OCD was not taken seriously and seen as less of a crisis than those who experienced such thoughts.

‘Do I need to turn up to A&E with blood pouring down my wrists? Because I hadn’t tried to kill myself I wasn’t being taken seriously. […] It’s almost like that question of “do you want to kill yourself?”, determines whether or not you get the help that you need, and it shouldn’t be with OCD. Like, if you are Googling for 8 hours a day and if you are afraid to go outside or you’re not speaking, within the OCD realm should be like that sort of tick.’ (W6)

#### Autonomy over the decision to be admitted

All eight women were admitted voluntarily to the MBU. One woman was later sectioned on the MBU (W7), and another was moved to a general psychiatric ward under a section of the Mental Health Act (W2).

Despite being voluntarily admitted, women felt as though they had no choice about admission. For some, this was because of the severity of symptoms and feeling as though they had run out of options. Some women described feeling forced into agreeing to informal admissions, owing to fears about what would happen if they did not comply). This was despite professionals reassuring them that they had autonomy over this decision.

‘I could voluntarily go into hospital and if I didn’t go in voluntarily, then they would have to section me. So as far as I understand, I was like, so there isn’t a choice.’ (W7)

‘They kept telling me it was a voluntary admission, and everyone kept reiterating that but, like it didn’t feel like that. I thought there was no other option.’ (W8)

### Theme 2: Developing a shared understanding of OCD

#### Sharing knowledge of OCD across the staff team

Both women and professionals discussed differences in OCD knowledge across the staff team. Women and professionals drew differences between permanent and temporary (or bank) staff, and between staff from different professional backgrounds. Clinical psychologists and psychiatrists were viewed as holding the OCD knowledge, and women highlighted a discrepancy between who holds this knowledge and who spends the most time with them. Women described interacting most with healthcare assistants (HCAs).

‘I didn’t experience anybody on there really knowing anything about it, apart from the psychiatrist who I saw once a week, who was…who was more like a visitor to the ward. So those people, those people that are dealing with you every day, that’s the people you know, they need to know.’ (W7)

Some women described how a lack of staff understanding about OCD led to them feeling as though they were being ‘stupid, ridiculous and irrational’ (W4).

Women and professionals discussed the importance of sharing OCD knowledge across the team. Professionals described attending teaching sessions and case discussions to distribute information about OCD. Clinical psychologists discussed taking a lead in sharing ‘formulations’, to support the team’s understanding of OCD and delivering training. All professionals voiced a desire for there to be ‘more training around about OCD for everybody’ (P2 – Occupational Therapist), however busy workdays and shift patterns made it difficult for universal attendance. Women highlighted the need for proactive training, rather than reactive in response to a woman with pOCD being admitted.

#### Piecing together an individualised understanding of OCD

Six women accessed individual sessions with psychologists during their admission. For most, these sessions involved learning about OCD.

‘They actually worked a lot about informing me about what OCD was. They showed me a lot of scientific literature about it, a lot of the evidence and you know, they found a case study about a woman who’d like, had almost exactly the same thoughts and things like that, so, like the science about these being terrible thoughts, and you won’t act on them.’ (W1)

Developing an understanding of OCD was viewed as important by both women and professionals, and key to understanding the rationale for exposure. This included thinking about ‘the history and the triggers’ (W3) and what was maintaining the OCD. Clinical psychologists referred to this as a ‘formulation’.

A proportion of women described experiencing co-occurring mental health difficulties, including depression and post-traumatic stress disorder (PTSD), and felt it was crucial to understand how these may be linked to OCD. Not understanding the ‘full picture’ (W2) was seen to hinder progress and in order ‘to treat those other difficulties effectively, the OCD can’t be ignored’ (W4).

This sub-theme draws on quotes from women and clinical psychologists only, which may reflect that a deeper understanding of OCD symptoms may not be shared across the wider MBU team.

#### Collaborative, transparent treatment plans

Where women felt there was a shared understanding of OCD between themselves and staff, this allowed for an ongoing dialogue between women and professionals. These women described the importance of knowing what was going to happen as part of their treatment and why this was happening, particularly regarding exposure work which involves exposure to feared stimuli.

However, many women did not understand why they were expected to complete exposure tasks. On reflection, women understood the rationale for this work; however, they felt this was not clearly explained at the time.

‘I was left to it all the time to just get on with this baby and couldn’t understand, I couldn’t understand why if I was feeling like I was gonna hurt her why I was being left to get on with it when there was other people there who seemed to be getting a lot more help.’ (W3)

In some instances, this resulted in friction between women and staff and a sense of unfairness among patients. In this example, there remains uncertainty around whether this was a planned task as part of OCD treatment, which she had not been informed about, or whether this occurred because this woman was being assessed as low risk, and staff resource being allocated elsewhere.

### Theme 3: A whole team approach to treatment

#### Taking responsibility for OCD treatment

There were differing views across professionals as to who holds the responsibility for treating OCD on MBUs, particularly supporting women with ERP tasks. Clinical psychologists described feeling as though this should be a shared responsibility among members of the team.

‘We might start but a lot of it would be followed up by the nurses or the assistant psychologists or the nursery nurses when they’re doing messy play and encouraging that that sort of mum to kind of sort of, stay with that anxiety.’ (P1 – Clinical Psychologist)

There was a recognition among clinical psychologists that therapy sessions only take up a small proportion of the week and other staff supporting this work outside of sessions offers an opportunity for learning to be reinforced. Other Multidisciplinary Team (MDT) professionals also recognised this but felt they had little guidance as to how to support women with pOCD. Supervision of indirect work was not specifically described by clinical psychologists, and some professionals saw OCD treatment as predominantly the job of clinical psychologists.

‘To be honest, we didn’t really… we wouldn’t usually get involved in any exposure work, like I knew it was going on but the women would just do that in their psychology sessions.’ (P8 – Nursery Nurse)

Some professionals described apprehension or reluctance to work closely with women with OCD. Professionals shared their doubts and fears about what may happen during exposure experiments. This appeared to be particularly present when working with women with intrusive thoughts of deliberately harming their baby.

‘I will absolutely say that it causes me, and I’d say others, anxiety sometimes when you think “is this really simply an OCD sort of thought?” […] you know is this woman going to be the one in 1000 who does act on it?’ (P6 – Occupational Therapist)

Women recognised this apprehension in professionals who they spent the most time with (e.g. HCAs) and described feeling as though these professionals were scared of them. This appeared to be particularly present for women who experienced intrusive thoughts of deliberately harming their baby.

#### Difficulty maintaining a consistent approach

Both professionals and women reflected on the challenges of working as a team to support women with OCD. Women noticed there was often a lack of consistency across the team, in terms of the support they received.

‘They kept changing tack as to like how much reassurance was OK to do and what wasn’t because they weren’t basing it off any kind of template or guide or advice or guidance about what was actually helpful or not helpful.’ (W7)

Similarly, strategies to manage anxiety were allowed by some members of the team but disallowed and removed by others. Professionals recognised that this inconsistent approach often ‘causes more distress’ (P4 – Ward Manager). Consistency was particularly challenging among bank staff, who had less of an awareness of care plans and women’s preferences.

Clear communication between professionals was seen as key to ensuring a consistent approach to supporting women. Handover was viewed by women as an opportunity for staff to agree how to best support women with OCD. However, often changes were not maintained over time or not implemented by all staff.

### Theme 4: Choice and control over exposure

#### Gradual and planned exposure

Most women had the opportunity to engage in exposure response prevention (ERP) tasks during their MBU admission. Collaboratively planning ERP tasks was important to all women. They valued being in control of what the exposure would be and when it would happen. Additionally, women expressed a preference for using a graded approach to facing their worst fears.

‘At first, I just had to be present when they bathed him, and then slowly I had to help and then eventually I had to like take over and be the one bathing him with one of them there.’ (W1)

Professionals also perceived grading exposure to be necessary, perhaps owing to the severity of OCD and concerns that exposure may be too distressing.

‘We set up, almost a kind of graded one, because the extreme of going from doing a nappy change with someone else to doing it alone in her room with the shutters closed was just too much for her.’ (P7 – Clinical Psychologist)

#### Unexpected, ‘non-therapeutic’ exposure

All women described encountering anxiety-provoking situations during their admission. This was experienced differently to planned ERP tasks, and it triggered high levels of anxiety for women. Women described feeling forced into terrifying situations and their anxiety was triggered during interactions with staff and other patients on the ward.

‘And when I came out to come and get him, they had…they’d just kind of done their own thing and just like gave him loads of toys that had literally just been in, like other baby’s mouth, and it just made the whole situation so much worse.’ (W2)

Additionally, illnesses spread among women and babies, which increased some women’s fears of contamination. Women who were frequently exposed to unexpected triggers described being unable to benefit from this exposure owing to high levels of anxiety.

‘I get the idea you expose yourself to the thing that frightens you and then gradually your anxiety will come down and you’ll be able to see the evidence that actually you know you’re OK […] But in that context, there was no come down. There was no way of coming down because there was no, I could not see any evidence […] The evidence was constantly there that it was a really dangerous place to be.’ (W7)

#### Meeting women halfway to manage distress on the ward

To manage unexpected exposure and the distress it can cause, all women described wanting to put in ‘boundaries’ (W4). For example, their baby using a separate playmat, staff not touching their belongings and eating separately. Women valued staff being open to making adjustments and being compassionate about the difficulty of encountering feared stimuli, although not all staff members did this.

‘[Nurse] said “oh I need you, I need to empty your bags and I need to go through everything.” And uh, for me from an OCD point of view, I was like, I do not want anybody touching… Thankfully the student nurse who was with her was like, “that’s OK, why don’t I do it with her?” And then she said to me […] “I don’t have to touch it. You can just show me everything”.’ (W4)

The majority of professionals also discussed the importance of meeting women halfway and trying to make the environment and interactions less distressing. This often meant women with OCD required a different approach to other women on the ward.

‘I think staff being open to doing things slightly differently to… not accommodate the compulsions, but to make challenging them a little bit easier erm rather than just boom, we’re gonna do things exactly the same as everyone else.’ (P9 – Psychiatrist)

Professionals also voiced the difficulty of trying to balance helping women to feel comfortable and less distressed, while also not feeding into the OCD.

### Theme 5: Ward as a safety net

#### Pushing with exposure experiments

Professionals who had supported women with exposure tasks viewed the ward as a ‘safety net’, containing their and the women’s anxiety about exposure tasks (P8 – Nursery Nurse; P3 – Clinical Psychologist). It was reassuring for staff to know that if women became overly anxious following exposure, members of staff would be available to support them to manage this. Psychologists described how the MBU environment allows them to have more control over designing ERP tasks, compared with in the community. Through controlling and managing the exposure, professionals were able to reduce the level of uncertainty, both for themselves and for women.

#### ‘Reassurance’ of observations

An aspect of the ward environment that provided professionals with a sense of safety was daily observations of women. Professionals found this particularly reassuring when they were worried that women might act on intrusive thoughts of harming their baby. However, for women with pOCD close observations may be unhelpful in the longer term.

Some women described observations initially reducing their worries about acting on thoughts of deliberate harm and fostering feelings of safety while on the ward. However, women reflected that leaving the ward and no longer being observed was scary as the observations had reinforced a belief that they needed to be observed in order to keep their baby safe and not act on their thoughts.

‘Suddenly you’ve gone from being watched all the time, checked on for every 15 minutes to being at home on your own and at first that didn’t feel safe and like I’d get really stressed out at home because you know my phone wasn’t charged or something didn’t happen or something changed.’ (W3)

### Theme 6: Transitioning back to ‘real life’

#### Translating learning from the MBU to home

There was a recognition among both women and professionals that the MBU environment is very different to home.

‘The worries and the fears that the person had on the ward weren’t relatable to the worries and the fears that they have at home out there and out and about in the real world.’ (P3 – Clinical Psychologist)

There was a belief among professionals and some women that this may limit the generalisability of learning from ERP tasks on the ward. Participants recognised the importance of spending time off the ward, practising exposure experiments in real life. For example, attending community baby groups, swimming, going to the shops and regular home leave.

For women experiencing intrusive thoughts of harming their baby, the MBU became a place of safety which initially reduced their worries but resulted in home leave feeling challenging and scary. Supporting women to access home leave may act as an exposure task, and support treatment. As with other exposure tasks, women had a preference for building up the amount of time they spent at home. However, for some women this was challenging because of the distance between the MBU and home.

#### Role of family in returning home

Women and professionals described partners and/or family members playing an important role in supporting women to implement learning from the MBU at home. One professional described offering psychoeducation to partners as ‘a really helpful approach’ to supporting the transition to home (P7 – Clinical Psychologist). However, most women’s families were not directly involved in psychology sessions and received updates at ward rounds or from women themselves. One professional identified this as an area for improvement for supporting women with OCD, owing to the role others can play in maintaining symptoms.

‘Thinking about how sometimes partners, fathers, significant others might be feeding into the OCD kind of cycle as well and might be unintentionally kind of maintaining that without realising, so more conversations around that with partners would be really helpful and also how they might be able to support that going forward.’ (P1 – Clinical Psychologist)

There was a recognition that not all women had family nearby and not all women had supportive familial relationships. For these women, ongoing community support was viewed as extremely important.

#### Plan for ongoing support

Women reported a range of outcomes following their MBU admission. Some experienced improvement in OCD symptoms. For others, symptoms worsened, particularly for those who did not access planned exposure work (W2 & W7). Regardless of reported symptom change, all women described ongoing difficulties at discharge.

Women and professionals advocated for ongoing mental health support in the community and viewed a clear plan for post-discharge support as essential. This was facilitated by good communication between MBU professionals and community teams and regularly inviting community professionals to ward rounds.

‘It was just reassuring to know that I would be seeing [care co-ordinator] and we had a couple of appointments booked in for when I left. I think the idea of going home was just so overwhelming but at least there was a bit of a plan.’ (W8)

Women reported wanting to continue psychology sessions and ERP tasks in the community. Both professionals and women recognised barriers to this, including there being no psychologists in some community teams. For one woman, who felt her community team forced her into the admission, it was difficult to work with members of the same team again after discharge.

## Discussion

In this study, we aimed to gain insight into the experiences of women with pOCD admitted to MBUs and professionals’ experiences of supporting women with pOCD in this environment. The research highlighted a number of challenges in providing treatment for pOCD in this environment.

Women and professionals viewed proactive and earlier support in the community as preferable to MBU admission, where possible. The findings suggest that in some areas of the UK, perinatal women remain unable to access timely evidence-based OCD interventions. This is concerning, particularly as distressing intrusive thoughts are commonly reported among women who attempt suicide in this period.^4^ All women reported ongoing pOCD symptoms at the point of MBU discharge, highlighting the need for ongoing psychological treatment in the community and the importance of responsive community perinatal teams that are well-equipped to support women with pOCD.

In line with best practice guidelines for OCD,^8^ most women accessed therapy involving ERP on the MBU, and this was viewed as a key part of treatment. However, some women did not access ERP during their admission, and many professionals were not involved in exposure work. There may be a number of reasons for this. Often admission to MBUs is unplanned or the length of stay is uncertain, both of which may have an impact on the provision and engagement with psychological therapy. Additionally, some women were experiencing thoughts of self-harm or ending their life, others experienced co-morbid PTSD which may have indicated other treatment options or may have overshadowed their OCD. MBUs offer a range of different interventions including pharmacological treatment, interventions to support the mother–baby relationship and occupational therapy, which may have been viewed as the most appropriate treatment for women at the time of their admission. Medication and specific mother-baby interventions were not prominent features of the current findings. While the current research did not specifically set out to explore women and professionals’ experiences of these interventions, this may also reflect women and professionals’ understanding of the OCD-specific aspects of treatment in comparison to the treatment women with other mental health difficulties access on MBUs and a desire to focus on these during interviews. The impact of uncertainty about admission length and the need to prioritise co-morbidities may not only be specific to women with OCD. Additionally, the escalation of safeguarding concerns in the context of maternal mental health difficulties may also be a shared experience among women with different diagnoses.

Additionally, professionals shared their apprehension about whether women may act on intrusive thoughts, which may have contributed to women not accessing ERP. This suggests there continues to be misunderstanding/doubt about pOCD diagnosis and risk.^26^ These worries may underpin a reluctance among staff to engage in exposure work,^11^ or a lack of confidence and guidance in how to support women with exposure tasks. Supporting MBU staff to facilitate exposure work outside of psychology sessions could provide women with the opportunity to access more intensive treatment, which has been found to be acceptable and effective treatment for pOCD.^14^

The current study highlights a distinction between planned ERP tasks and unexpectedly encountering anxiety-provoking situations on the ward. In CBT, therapeutic exposure is conceptualised as agreed, pre-planned exposure tasks while not carrying out related behavioural/mental compulsions. When women encountered anxiety-provoking situations unexpectedly, they described frequently using behaviours to try and manage their anxiety which may have contributed to these triggering experiences feeling less therapeutic. Women valued being able to exert control over what they were exposed to on the ward, and how this exposure took place. Within cognitive theory, the strategies women used to limit exposure and manage their anxiety (e.g. using a separate playmat for their baby) may be conceptualised as safety behaviours, in that they reduce anxiety in the short term. However in the longer term they may reinforce women’s beliefs that they must do these behaviours in order to be safe.^33^

Within CBT, individuals are typically encouraged to drop all safety behaviours during exposure tasks.^34^ However, owing to the severity of women’s OCD, not using any safety behaviours on the ward was viewed by women and professionals as too overwhelming for women. The use of such behaviours appeared to play a role in helping women engage in activities on the ward, in keeping with research that has found carefully using safety behaviours can support engagement with exposure tasks.^35,36^ This was also seen in planned exposure experiments, where women and professionals described the importance of using hierarchies to grade and build up to feared stimuli. As there are nuanced differences between when a behaviour is helpful in promoting engagement and when it is counter-therapeutic,^37^ there needs to be a collaborative and clear plan between women and staff about which behaviours are helpful to use and in which circumstances. Additionally, collaborative and clear planning may support women to engage in exposure tasks and support the developing relationship between women and professionals.^12^

There were differences in how the ward was experienced by women with different OCD presentations. Women who reported intrusive thoughts about contamination, generally experienced the ward as a less safe place than women with thoughts of deliberate harm. This is perhaps unsurprising given the nature of the MBU environment and shared facilities. In contrast, women with thoughts of deliberately harming their baby described generally feeling safe on the ward. High staff presence and observations appeared to reassure women they would not act on these thoughts. However, in the longer-term women did not learn that staff were not needed for them to be safe. Close observations and the reassurance they imply could therefore be conceptualised as a safety behaviour, suggesting their usage needs to be considered carefully for each woman admitted with pOCD to ensure this is not hindering long-term improvement in OCD symptoms.

Within CBT for OCD a shared and individualised formulation should underpin therapy.^38^ Sharing formulations with the team in an in-patient context could promote a shared understanding and personalised approach to treatment, reducing the likelihood of misunderstandings between women and staff. For example, adapting certain ward procedures such as observations and bag searches. Of note, only psychologists used the terminology ‘formulation’, suggesting that this may not be a shared concept among women and the MBU team. This use of psychological jargon may be a barrier to developing a shared understanding of OCD on the MBU.

Findings highlighted the importance of professionals maintaining a consistent approach to supporting women with OCD on MBUs. This requires a shared understanding of the treatment plan and effective communication between staff, in order for all professionals to be aware of an individual’s treatment stage and receive regular updates on treatment progress. Rotating staff teams, shift work and the presence of bank staff were noted to lead to inconsistencies in care. This is a recognised difficulty in treating OCD on general in-patient wards.^16^

Previous qualitative studies exploring women’s experiences of MBU admission have not disaggregated data by specific diagnosis;^22^ however, figures suggest that women with pOCD made up a small percentage of these samples. Similarly to previous qualitative studies, the relationship with staff was also important to women with pOCD and the current findings echoed the importance of staff understanding women’s difficulties, and showing empathy in response to distress.^22,23^

Further to this, it is challenging to draw comparisons between the current findings and outcomes for individuals with non-perinatal OCD who have experienced admission to general psychiatric wards, as there is limited outcome data available.^16^ Research exploring the outcomes of specialist OCD residential units, has found significant decreases in OCD symptoms following a residential stay.^17^ This is likely a different setting to an MBU, as clinicians have specialist OCD knowledge and this is the primary focus of treatment. To date, research has not specifically explored individuals’ experiences of these programmes.

### Key clinical implications

The findings point towards a clear need for clinical guidelines for supporting women with pOCD on MBUs and provide areas for consideration in the development of such guidelines (Table 4).

**Table 4:** Key clinical implications

Clinical implication	Further details
1. Regular obsessive–compulsive disorder (OCD) training	Regular OCD training is needed for the whole staff team. This may include psychoeducation about perinatal OCD, discussion of case examples and role playing how to support with exposure tasks.
2. Clear communication	Clear communication across the Mother and Baby Unit (MBU) team. Multiple methods of communication are needed, to ensure all staff (including bank staff) receive the same information in order to provide a consistent approach to care. Shared, collaborative and specific treatment plans that are regularly reviewed.
3. MDT involvement in exposure and response prevention (ERP) tasks	MDT to support women to engage in ERP tasks outside of psychology sessions. This will require good communication across the team and clear treatment plans.
4. Frequent home leave	Frequent home leave is needed to offer women the opportunity to generalise their learning from exposure tasks on the ward, by carrying out tasks at home and in their local community.
5. Involvement of partners and/or family members,	Involvement of partners and/or family members, where appropriate and with women’s consent. Partners and/or family members should be explicitly involved in perinatal OCD treatment on the MBU and provided with psychoeducation. Video conferencing may facilitate the inclusion of partners and family members.
6. Responsive support from community perinatal mental health teams	Responsive support from community perinatal mental health teams that are well equipped to offer evidence-based OCD treatment. Further investment is needed to ensure there is equitable access across the UK, both prior to and following MBU admission.

MDT, Multidisciplinary Team.

### Limitations

A purposive sampling strategy was used to capture the experiences of different professional groups, with differing amounts of MBU experience. It was challenging to recruit HCAs and bank staff, perhaps because of shift patterns and having limited time to participate. Considering the differences identified between staff, future research should focus on HCAs and bank staffs’ experience to inform the development of training for these professional groups.

Participants in the study had experience of 8 MBUs, out of the 22 units across the UK. The frequency of OCD presentations on MBUs may vary nationally, owing to differences in bed numbers and the proximity of MBUs to one another. Therefore, professionals’ familiarity, knowledge and awareness of pOCD may also vary which may have influenced results in unknown ways.

Women were largely from White British backgrounds. Because of limited research exploring pOCD on MBUs, it is unclear whether this is representative of women with pOCD admitted to MBUs across the UK. Additionally, there is limited research exploring the experience of pOCD across diverse groups. As research develops, it will be helpful to establish the demographic characteristics of women with pOCD admitted to UK MBUs. There was limited background or qualitative data on comorbidities and medication in this study.

## Supporting information

Davenport et al. supplementary material Davenport et al. supplementary material

## Data Availability

Data is qualitative and we do not have ethical permission to share.

## Footnotes

a. A note on language: Perinatal mental health research to date has predominantly recruited cis-gender women (those who identify as female and were assigned female at birth). This is reflected in the gendered language used within clinical guidelines and services (e.g. Mother and Baby Units). The research team strongly supports the use of gender-inclusive language and exploration of diverse experiences; however, in this research and the perinatal research that is referenced, all participants identified as women, and therefore the decision was made to use the terminology of ‘women’ and other terms such as mother and maternal.

## Supplementary material

The supplementary material is available online at https://doi.org/10.1192/bjo.2026.10976
