# Supplementary material for: The treatment experiences of women with perinatal OCD on Mother and Baby Units: qualitative investigation of the perspectives of women and professionals
Source: BJPsych Open. 2026 Feb 10;12(2):e61. doi: 10.1192/bjo.2026.10976 (PMC12926895; doi:10.1192/bjo.2026.10976)
Supplement: Davenport et al. supplementary material [file S2056472426109764sup001.docx]

Interview Topic Guide – Service Users

**Interview Schedule**

**MOTHERS**

**Main reason for admission and circumstances under which participants were admitted**

- Could you tell me about how you came to be admitted to the MBU?
- Was this a voluntarily admission or were you admitted under the Mental Health Act?
- What was your experience of recognising that you had OCD - was this something that you knew about before having your baby or something that occurred during the perinatal period?
- How did you come to be diagnosed with OCD?
- Were there any issues with recognising you had OCD either from your point of view, or from the point of view of professionals?
- What were the main difficulties troubling you at the time of admission?
  - Prompt: For some people with OCD they may experience thoughts about contamination, other people may experience thoughts about harm coming to their baby.

**Experience on the MBU**

Opening question

What was your experience of being on the mother and baby unit?

**Staff**

- What was your experience of working with different members of staff on the unit?
  - What type of staff did you come into contact with on the unit (e.g. Nursery Nurses, Occupational Therapists)?
  - Did you and the staff on the unit all have a shared understanding of the OCD or were there differences between the different professions?
  - Did it feel as though all of the staff team had a consistent approach to your treatment or were there differences between the different professions?
  - From your perspective, how were these differences managed?

**Specific treatment on the MBU for perinatal OCD**

- Whilst you were on the unit what treatment did you receive?
  - Example prompts: Medication, support for developing relationship with baby, groups, parent-infant Psychotherapy?
  - Did you find the elements of the treatment helpful?
- Did you receive specific treatment for perinatal OCD?
  - Did you receive Cognitive Behavioural Therapy (CBT) on the unit?
  - How many sessions of CBT did you have?
  - Who delivered this treatment?
  - What would you say was the main focus of CBT sessions?
    - *Prompts: Did you do any experiments in sessions? What did these involve?*
  - Did CBT sessions impact your day-to-day behaviour on the unit? If so, how?
  - Was your partner and/or family involved in this aspect of the treatment?
    - How did you find this?
  - How helpful did you find CBT?
    - What was most/least helpful about CBT?
  - How did this fit in with the overall treatment package you received on the unit?
- Had you received treatment from mental health services previously (for OCD or for other mental health difficulties)?
- If yes, how was treatment the same/how did it differ?

**Baby**

- Did you feel supported in caring for your baby by the staff?
- What impact, if any, do you think the admission may have had on your baby?

- Did your admission impact your relationship/bond with your baby, and if so, in what way?

**Other patients**

- What was your experience of interacting with other Mothers on the unit?

**Inclusion of partners and/or family members in treatment**

- From your perspective, what was your admission like for your family?
- Did you find that your partner and/or family members were included in your overall treatment on the MBU?
- If yes:
  - What was helpful about this?
  - Was there anything less helpful about including your family members in treatment?
- If no:
  - Do you think this is something you would have found helpful?
  - If so, why?
- How did you find home visits/home leave?

**Social services**

- Was there a social worker on the unit? What was your level of interaction with them?
- Did the unit make a referral to children’s social services?
  - If so, what was your experience of this process?

**Discharge**

- How did you find the process of discharge from the unit and returning home?
  - What, if any, support did you receive after discharge from the MBU for perinatal OCD?
  - Did you receive support from another mental health service? If yes, what was your experience of this?
- How do you think women with perinatal OCD could be best support after discharge from MBUs?

**Overall Satisfaction**

- What did you find most helpful about your admission to the MBU?
- What did you find least helpful about your admission to the MBU?
- What impact do you think your admission had on your mental health?
  - Did you see a reduction in your OCD symptoms?
- Is there anything about your experience on the MBU that I haven’t asked about, but you think it’s important for us to know?

**Suggestions for improving MBUs for mothers with perinatal OCD**

- Based on your experience what changes would you like to see on MBUs?
- How could MBUs better support women with perinatal OCD?

Interview Topic Guide - Professionals

**Interview Schedule**

**PROFESSIONALS**

**General experience**

- What has been your experience of working with women with OCD on the MBU?

**Number of women with perinatal OCD on MBUs**

- In your experience, is it common for women with OCD to be admitted to MBUs?
- Under what circumstances are women with OCD admitted to MBUs?

**Recognising perinatal OCD on MBUs**

- How do professionals identify perinatal OCD on MBUs and distinguish perinatal OCD from other perinatal mental health difficulties?
  - Is this ever challenging?
- What are the main types of OCD that you have come across on MBUs?

**Participants’ experience of working as part of a multidisciplinary team**

- Which other professionals do you/have you worked with on units?
- What is your experience of working as part of a team on MBUs, particularly when working with women with perinatal OCD?
  - Helpful aspects of working with other professionals?
  - Are there any aspects of team working that are challenging with regards to supporting women with perinatal OCD?
  - How have you and the team managed/resolved these difficulties?

**Current theoretical understanding of perinatal OCD on MBUs**

- How do/did you and your colleagues on the MBU understand what/conceptualise perinatal OCD is?
  - What is your understanding of what keeps perinatal OCD going/prevents improvement in symptoms?
- Is this understanding shared amongst the wider team and other professional groups?
  - Does it feel as though there is a shared understanding amongst the team?

**Treatment currently offered on MBUs for perinatal OCD**

- How have you supported women with perinatal OCD on the unit?
- What treatment are women with perinatal OCD typically offered on MBUs?
- Have you seen CBT be offered to women with perinatal OCD/have you delivered CBT for perinatal OCD on MBUs?
  - If participant has delivered CBT:
    - What does this typically involve?
    - How successful has CBT been for perinatal OCD on MBUs?
      - Was there anything about the MBU environment that facilitated CBT for OCD?
      - Was/is there anything about the MBU environment that made delivering CBT for OCD more challenging?
      - Are family members typically involved within CBT sessions? Is this helpful/what is less helpful about this?
      - How does CBT fit within the wider treatment package for women with perinatal OCD on MBUs?
  - If participant has not delivered CBT:
    - From your perspective, what does CBT usually involve for women with OCD?
    - How helpful is CBT for women with perinatal OCD on MBUs and in what way is it helpful?
    - How does CBT fit within the wider treatment package available on MBUs?
  - In your experience, why might CBT not be offered to someone with perinatal OCD on the MBU?
- What other treatments are offered to women with perinatal OCD?
  - What other treatments do women with perinatal OCD receive on MBUs?
    - Medication?
    - Groups?
    - Support for developing relationship with infant?

If participants involved in delivering a particular treatment can ask follow up questions:

- Do you find this helpful for women with perinatal OCD?
  - Prompt: In what way?

**Inclusion of partners and/or family members in treatment**

- How can family members support treatment?
- In your experience, have family members or partners been included in women’s treatment?
- Have there been any barriers to including family members?

**Managing risk associated with perinatal OCD**

- In your experience, what issues around risk have you encountered when working with women with perinatal OCD, on MBUs?
- How is this risk understood by the wider team? Is there a consistent understanding amongst the team?
- How are these risks managed on MBUs?
- Do risk issues and the subsequent management of these risks differ depending on the type of OCD a woman is presenting with?

**Discharge**

- How do you think women are/would be best supported after discharge from the MBU?

**Suggestions for improving MBUs for mothers with perinatal OCD**

- - What works particularly well for women with perinatal OCD on MBUs?
  - How could MBUs better support women with perinatal OCD?
  - Are there any changes would you like to see on MBUs?
